# High gene flow and lack of genetic structure in the commercially important crab *Cancer porteri* (Brachyura: Cancridae) along 1,500 km of the Chilean coast revealed by SNP markers

**DOI:** 10.7717/peerj.20727

**Published:** 2026-01-22

**Authors:** Juan Soto, Noemí Rojas-Hernández, Caren Vega-Retter, Luis Miguel Pardo, Carolina Parada Veliz, María de los Ángeles Gallardo Salamanca, David Veliz

**Affiliations:** 1 Departamento de Ciencias Ecológicas, Facultad de Ciencias, Universidad de Chile, Santiago, Región Metropolitana, Chile; 2 Instituto de Ciencias Marinas y Limnológicas (ICML), Laboratorio Costero de Recursos Acuáticos de Calfuco (LCRAC), Facultad de Ciencias, Universidad Austral de Chile, Valdivia, Región de Los Ríos, Chile; 3 Centro de Investigación Dinámica de Ecosistemas Marinos de Altas Latitudes (IDEAL), Fondo de Financiamiento de Centros de Investigación en Áreas Prioritarias (FONDAP), Valdivia, Región de Los Ríos, Chile; 4 Departamento de Geofísica, Universidad de Concepción, Concepción, Región del Biobío, Chile; 5 Center for Ecology and Sustainable Management of Oceanic Islands (ESMOI), Departamento de Biología Marina, Facultad de Ciencias del Mar, Universidad Católica del Norte, Coquimbo, Región de Coquimbo, Chile

**Keywords:** *Cancer porteri*, Genetic structure, Gene flow, Single-nucleotide polymorphisms, Marine connectivity, Planktonic larval duration, Chilean coast

## Abstract

Studying the distribution of genetic diversity and connectivity patterns is crucial for understanding the ecology of marine species and informing fisheries management decisions. Despite the heterogeneity of the Chilean coast, characterized by upwelling zones and biogeographical breaks, specific benthic species display high genetic homogeneity, likely due to high migratory flow facilitated by long planktonic larval duration (PLD). In Chile, the artisanal fishery targets various crustacean species, with the crab *Cancer porteri*, commonly known as “Jaiba Limón” or lemon crab being one of the important species representing 17% of total brachyuran landings in the last decade. In this study, the population structure, genetic diversity, and gene flow of *C. porteri* were analyzed, using data from seven different sample sites along 1,500 km of the Chilean coast from two samples in 2014–2015 and five in 2023–2024. Based on variability at 3,532 single-nucleotide polymorphisms (SNPs) in 127 individuals, the results revealed stable genetic diversity in space and time, and a high effective population size, with no evidence of genetic structure among sampling sites. The results suggest that *C. porteri* constitutes a single large genetic population across the area between 23°37′S and 36°36′S, with high gene flow among sites in both temporal periods. This lack of genetic structure appears to result from the high gene flow among all studied locations, as observed in other cancrid species in the same area. The long PLD, high fecundity and strong offshore advection capacity may contribute to their high dispersal potential, being an important precedent for future management plans for the species, which should also be complemented with studies that better describe demographic and biological aspects of the species.

## Introduction

Connectivity among marine populations, defined as the exchange of individuals and the resulting gene flow between them, can determine colonization and repopulation processes and alter the patterns of genetic diversity that exist within populations, directly affecting their capacity to cope with local extinction, adaptation to environmental changes, and fishing pressure (Selkoe et al., 2016; Chase et al., 2020). Connectivity patterns are fundamental for the establishment of marine protected areas, management of fish stocks, and conservation efforts (Treml et al., 2015; Fontoura et al., 2022). For example, considering connectivity patterns in Cumulative Impact Assessment models of the blue mussel *Mytilus edulis* shows that local pressures can impact a considerably larger and more distant area, with an impact of 20–30% in some small localities (Jonsson et al., 2021). In benthic invertebrates, connectivity depends mainly on the dispersal ability of their larval stages, due to the limited or non-existent movement of their adult phases (Cowen & Sponaugle, 2009). Oceanographic factors such as the direction and strength of marine currents, coastal upwelling, tidal movements, water stratification, coastal topography and physicochemical conditions collectively facilitate connectivity between distant areas or generate local larval retention zones (Cowen et al., 2007; Gawarkiewicz, Monismith & Largier, 2007; Teske et al., 2016).

Furthermore, biological factors can also affect population connectivity. Larval behavior, such as vertical migration to avoid predators during periods of high visibility, affects the survival and movement of larvae, since the speed and direction of currents vary according to depth (Bandara et al., 2021). However, one of the most important biological traits affecting dispersal ability is the time the larvae spend in the plankton while completing their larval development, known as planktonic larval duration (PLD), a widely used metric for modeling marine population connectivity that has a strong effect on metapopulation stability (Bani et al., 2019). Studies described that PLD is one of the most significant predictors of the geographical distribution of marine species, even more important than adult characteristics (Macpherson & Raventos, 2006; Barroso et al., 2022). Consequently, the interaction of oceanographic and biological factors influences the dispersal capacity of the larvae.

The marine environment is highly heterogeneous, posing challenges for the study of dispersal patterns and making it difficult to predict larval trajectories (Gawarkiewicz, Monismith & Largier, 2007). In recent years, genomic tools have profoundly enhanced the understanding of genetic heterogeneity in marine populations, enabling inferences to be made about connectivity, larval dispersal and gene flow between populations (Selkoe et al., 2016). Studies indicate that the levels of genetic structure observed in various invertebrate species correlate with their dispersal capacity. Species with short larval development periods (<1 month) show patterns of genetic structure that coincide with the biogeographic barriers described for the Chilean coast and areas of high environmental heterogeneity (Barahona et al., 2019; Haye et al., 2019; Quesada-Calderón et al., 2021; Saenz-Agudelo et al., 2022; Peluso et al., 2023). In contrast, species with long larval periods (>1 month) appear to be less sensitive to these barriers and present less genetic structure (Haye et al., 2014; Rojas-Hernandez et al., 2016; Veliz et al., 2022; Tubin-Arenas et al., 2025).

*Cancer porteri* Rathbun, 1930 (Brachyura: Cancridae) is a decapod of commercial importance for artisanal fisheries in central and central-south of Chile and constitutes part of the bycatch fauna in demersal crustacean fisheries off north-central and south-central Chile. Its distribution in the southern hemisphere ranges from Isla Lobos de Afuera, Peru (6,9°S), to Mocha Island, Chile (38,4°S) (Inostroza et al., 1982; Carvacho, 1989) and it is distributed at depths ranging from 0 to 375 m (Henríquez & Bahamonde, 1976), preferring muddy bottoms (Jesse & Stotz, 2002). In Valparaíso, Chile, females reach sexual maturity at a cephalothorax width of 92 mm (Inostroza et al., 1982), which corresponds to sexual maturity at 2.3 years for females, and 2.5 years for males (IFOP, 2017). Females have high reproductive potential of 288,750 to 323,130 eggs in females with a cephalothorax length of 61.3 to 64.9 mm (Antezana, Fagetti & López, 1965), although fecundity up to 740,920 eggs on females of 83.8 to 118.2 mm was reported by Moreno (1989). Its larval development has been described only up to the first zoea stage (Fagetti, 1960), but other species of the family Cancridae in Chile present PLD > 2 months, for example, *Romaleon setosum* with 84 days at 16 °C (Weiss et al., 2009) and 60 days at 15 °C in *Metacarcinus edwardsii* (Quintana, 1983). Similarly, in the Northern Hemisphere, the Dungeness crab *Cancer magister* presents a PLD of approximately 80 days until the molt to the megalopa stage (Rasmuson, 2013).

In Chile, *C. porteri* is extracted from the Valparaíso region (32°02′S) to the Biobío region (36°49′S), with a total landing of 446 tons in 2023 (Sernapesca, 2023) and representing 17% of total brachyuran landings in the last decade (2009–2019) (Ávila-Thieme et al., 2025). Despite the species’ importance to artisanal fisheries, there are significant gaps in the biological knowledge of this crab. The latest effort to update reproductive and growth parameters was carried out by the Fisheries Development Institute of Chile (IFOP, 2017), but information on the genetic diversity and population genetic structure is currently unavailable.

This study aims to describe the population genetics of *C. porteri* along 1,500 km of coast, with estimation of genetic diversity indexes, genetic structure and gene flow between Antofagasta (23°37′S) and Tomé (36°36′S), Chile, with samples from two periods 10 year apart (2014–2015 and 2023–2024) to assess for temporal changes in the genetic structure. Because other species of the Cancridae family have larval development periods of more than 1 month, we expect results similar to those observed in *M. edwardsii* (Veliz et al., 2022), with the presence of a panmictic population with high gene flow between the sampling sites and without changes over time.

## Materials and Methods

### Sampling sites

**Figure 1:**
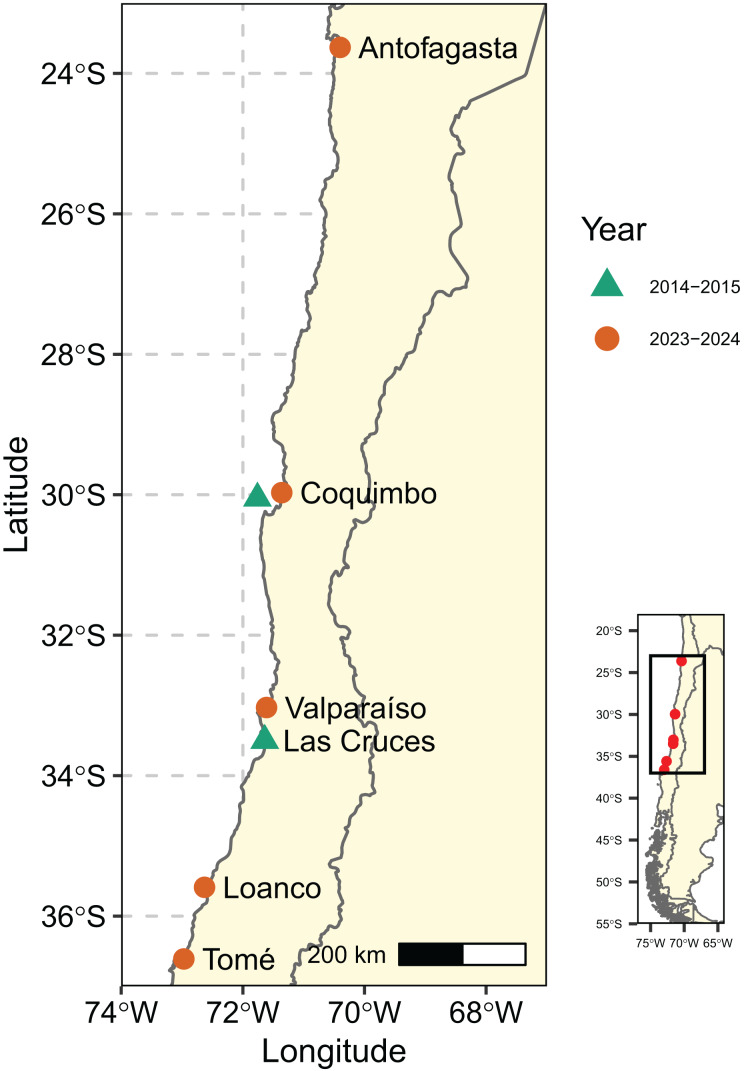
Samples sites of *Cancer porteri*. Green triangles represent samples from 2014–2015, and orange circles represent samples from 2023–2024.

Samples from 161 individuals were used in this study. Some of the samples were obtained in 2014–2015 Coquimbo (four) and Las Cruces (30), while the rest were sampled in 2023–2024 from Antofagasta (11), Coquimbo (28), Valparaíso (32), Loanco (39) and Tomé (17) (Fig. 1). Crabs were obtained by local fishermen using traps, the geographic assignment of each sample was therefore based on the fishermen’s known fishing areas within each locality. Taxonomical identification was done on site, and a pereiopod from each specimen was preserved in 95% ethanol. Sample collection was approved by the Institutional Committee for the Care and Use of Animals (CICUA) of Universidad de Chile (code 20379-FCS-UCH). All samples from Antofagasta and two from Coquimbo (one from 2009 and the other from 2015) were obtained from the Biological Collections Room of the Universidad Católica del Norte (SCBUCN) and originate from trawl net sampling. Tissue was extracted from the pereiopods by dissecting between the segments to avoid damaging the specimens, which are preserved in 96% ethanol. For Coquimbo, only the sample from 2015 passed single-nucleotide polymorphism (SNP) filtering. Catalog numbers for samples obtained from the SCBUCN collection are indicated in Table S1.

### Sequencing and SNP calling

From each pereiopod, a small sample of muscle tissue was sent to Dart Diversity Arrays Technology Pty Ltd. (DArT, Canberra, Australia) to perform DNA extraction and parallel sequencing. The library preparation process is detailed by Kilian et al. (2012). Briefly, the genome complexity is reduced by digesting the DNA with the restriction enzymes *Pstl* and *HpaII*. Then, fragments of >200 bp are ligated with an 8 bp barcode and amplified by PCR. Sequencing was performed in an Illumina NovaSeq X+ sequencer, demultiplexing and DNA barcode removal was performed in Dart to proceed to SNP detection.

Using the dartR package version 2.9.7 (Gruber et al., 2018; Mijangos et al., 2022) in R 4.4.1 environment (R Core Team, 2024), the SNPs were filtered to: eliminate secondary SNPs, retain loci with read depths between 5 and 200 to remove low confidence SNPs and excessively covered loci that could represent sequencing artifacts, and retain loci with >99% reproducibility. Monomorphic loci also were eliminated. Subsequently, a filter command line was performed to eliminate loci and individuals with missing data. First, we eliminated individuals with a call rate below 70% and loci with a call rate below 90%. Then, we applied another filter to eliminate individuals with a call rate below 85%. All SNPs with a minor allele frequency (MAF) <1% were eliminated. The genomic relatedness matrix showed that several pairs of individuals from Las Cruces 2014 and a pair from Coquimbo 2015 presented a high level of relatedness, thus we discarded one individual of the pair for the database used in the next analyses.

### Population genetic structure, genetic diversity, and contemporary gene flow

The data was processed as previously described in Veliz et al. (2022). Specifically, for population structure analysis all variants showing signals of selection were eliminated to avoid bias in the estimation of differences between sampling sites. For this, three methods were used to detect loci under selection. First, the outFLANK method implemented on dartR package based on the F_ST_ distribution of the loci (Whitlock & Lotterhos, 2015). Second, the relationship between F_ST_ and heterozygosity implemented in the Fsthet package version 1.0.1 (Flanagan & Jones, 2017). Both methods are implemented on R software. Third, the Bayesian method implemented in the BayeScan 2.1 software (Foll & Gaggiotti, 2008). Thus, we erased all loci identified with the three methods used.

Loci with significant deviations from Hardy-Weinberg equilibrium (*p* < 0.05 using the false discovery rate or FDR) identified with the dartR package, as well as loci showing significant linkage disequilibrium (LD) using the PLINK 2.0 software (Chang et al., 2015) (window size of 50 variants, step of 10, and a LD threshold of 0.5) were excluded. The remaining loci, after all these filters steps, comprised the set of neutral SNPs used in the subsequent analysis.

Genetic diversity was assessed using the package diveRsity (Keenan et al., 2013), comparing expected (H_E_) and observed (H_o_) heterozygosity levels, inbreeding coefficient (F_IS_), and allelic richness (AR) among locations. Population genetic structure was analyzed using three methods: (i) principal coordinates analysis (PCoA), (ii) a pairwise F_ST_ for which significance was obtained with 1,000 permutations (both methods are implemented in the dartR library) And, (iii) a Bayesian approach using the STRUCTURE software (Pritchard, Stephens & Donnelly, 2000) to search for the most probable number of genetic clusters (K). The procedure was run five times for each K (from K = 1 to K = 7) with a burn-in of 100,000 and an after-burn-in of 1,000,000 iterations each. The best K was estimated in the StructureSelector platform (Li & Liu, 2018) using the posterior probability (mean LnP(K)) (Pritchard, Stephens & Donnelly, 2000) and the bar plots for the individual assignment to each K group. Contemporary effective population size (*N*_*e*_) was determined for each genetic population and year in NeEstimator v2.x (Do et al., 2014) using the linkage disequilibrium method (Waples & Do, 2008) and assuming random mating. Reported results correspond to estimation excluding low frequency alleles (0.05) for bias correction (Waples, 2024).

For gene flow analysis, the software EEMS (Estimating Effective Migration Surfaces) was used to visualize gene flow patterns from the georeferenced samples using variation in effective migration across the habitat (Petkova, Novembre & Stephens, 2016). EEMS was run after separating the 2014–2015 and 2023–2024 data sets (2,196 and 3,513 neutral SNPs, respectively). The method infers migration parameters that best reproduce the spatial genetic structure observed in the dataset under a stepping-stone framework. The resulting estimates are spatially interpolated to generate an effective migration surface, which visually depicts relative gene flow patterns across the landscape. Areas of high historical connectivity are shown in blue, whereas zones with low gene flow appear in orange (Petkova, Novembre & Stephens, 2016). EEMS analyses were conducted as described by Veliz et al. (2022), with 500 demes and three independent Markov chain Monte Carlo runs, each comprising 5,000,000 iterations. The first 1,000,000 iterations were discarded as burn-in, and posterior samples were retained at intervals of 9,999 iterations. Proposal variances were tuned to maintain acceptance rates between 10–40%. Visualization of effective migration surfaces was performed in R using the rEEMSplots2 package (https://github.com/dipetkov/reemsplots2). All maps were made using the R package rworldmap (South, 2011).

Finally, historical migration rates were calculated with the MIGRATE software (Beerli & Felsenstein, 2001), which employs a coalescent approach to estimate mutation-scaled migration rates (*M*) between each group during the last 4N_E_ generations. The software was run for the 2014–2015 and 2023–2024 data sets as described by Veliz et al. (2022), with default settings except for: (a) one single long run utilizing heating with temperatures of 1.0, 1.5, 3.0, and 1,000,000; (b) 1,000,000 genealogies were run with a sample increment of 10; and (c) discarding as burn-in the first 100,000 genealogies. The uniform prior distribution was used for *Θ* (from 0 to 0.1) and *M* (from 0 to 100,000). We tested three models that represent the probable gene flow in the studied area: (a) panmixia model, (b) full migration, and (c) directional migration towards the north (northward). To identify the best model, each analysis was performed twice for two independent subsets of 1,000 SNPs, using the bf.py Python script provided by Beerli et al. (2019) that compares the Bezier log marginal likelihood values obtained in each model. The software STRUCTURE and MIGRATE were run on Cyber Infrastructure for Phylogenetics Research (CIPRES) (Miller, Pfeiffer & Schwartz, 2010).

## Results

A total of 43,976 SNPs were recovered. After filtering, this was reduced to 3,532 neutral loci in 127 individuals. The final number of samples per location is shown in Table 1. Genetic diversity parameters were similar across sample sites and year, with minor differences likely influenced by the small sample size of the 2014–2015 samples (Table 1). Allelic richness was lower in Antofagasta and Coquimbo 2015, with 1.12 and 1.09, respectively, the two locations with the lower sample size. The same was observed with heterozygosities. Inbreeding coefficient (F_IS_) showed higher values in Antofagasta (0.276) and Valparaíso (0.227), and lower in Loanco (0.171).

**Table 1:** Genetic diversity parameters per location and year.

Location	Year	N_(i)_	N_(f)_	AR	Ho	He	uHe	F_IS_
Antofagasta	2023	11	9	1.12	0.060	0.078	0.083	0.276
Coquimbo	2014–2015	4	2	1.09	0.070	0.064	0.087	0.205
Coquimbo	2024	28	27	1.16	0.076	0.091	0.092	0.177
Valparaíso	2023	32	19	1.14	0.071	0.090	0.093	0.227
Las Cruces	2014	30	16	1.16	0.077	0.093	0.097	0.200
Loanco	2023	39	38	1.16	0.076	0.091	0.092	0.171
Tomé	2024	17	16	1.15	0.077	0.091	0.094	0.178

**Note:**

N_(i)_, initial number of samples; N_(f)_, number of samples after filtering; AR, allelic richness; Ho, observed heterozygosity; He, expected heterozygosity; uHe, unbiased expected heterozygosity (corrected for sample size); FIS, inbreeding coefficient.

Six out of 21 pairwise comparisons of F_ST_ showed statistical significance (*p* < 0.05). There was no significant F_ST_ value between the two sample sites from 2014-2015 (Table 2). Between the two temporal samples, Las Cruces presented significant F_ST_ with Antofagasta and Coquimbo 2024 (both <0.001), and Coquimbo 2014–2015 showed significant F_ST_ with Coquimbo 2024 (0.042), although there may be an individual-specific effect given the low sample size in Coquimbo 2014–2015. Between the locations sampled in 2023–2024 the *p*-value was significant between Coquimbo 2024 and Valparaiso (*p*-value = <0.001), and between Antofagasta and all sites from Valparaíso to the south (all <0.001).

**Table 2:** Genetic differentiation between sample sites. Pairwise fixation index (F_ST_) is specified under the diagonal and Bonferroni corrected *P*-values are above the diagonal.

FST\*p*-value	ANT 2023	COQ 2015	COQ 2024	VAL 2023	LCR 2014	LOA 2023	TOM 2024
ANT 2023	–	0.105	0.999	<0.001	<0.001	<0.001	<0.001
COQ 2015	0.0342	–	0.042	0.336	0.147	0.063	0.252
COQ 2024	0.0026	0.0301	–	<0.001	<0.001	0.084	0.105
VAL 2023	0.0127	0.0223	0.0044	–	0.021	0.756	0.231
LCR 2014	0.0105	0.0250	0.0052	0.0041	–	0.042	0.999
LOA 2023	0.0091	0.0284	0.0022	0.0016	0.0027	–	0.999
TOM 2024	0.0111	0.0238	0.0032	0.0031	0.0021	0.0010	–

**Note:**

ANT, Antofagasta; COQ, Coquimbo; LRC, Las Cruces; VAL, Valparaíso; LOA, Loanco; TOM, Tomé.

**Figure 2:**
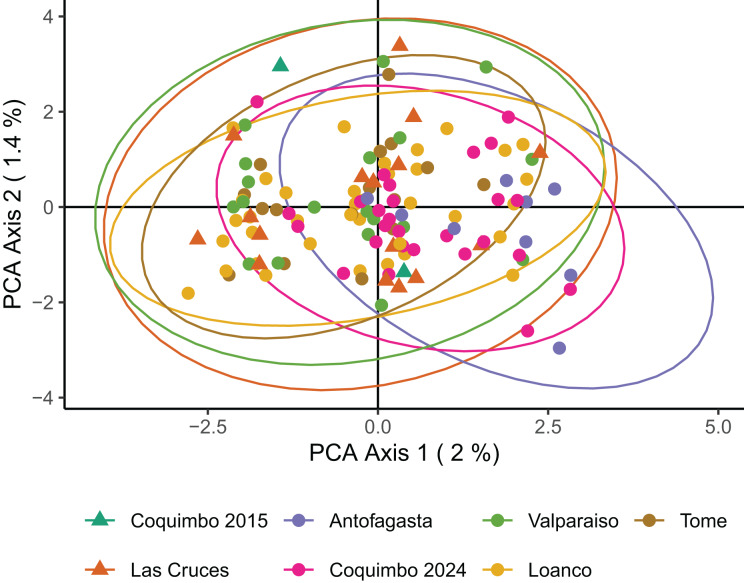
Principal coordinates analysis (PCoA) on 3,532 neutral SNPs of *C. porteri*. Triangles represent samples from 2014–2015, and circles represent samples from 2023–2024. Colors correspond to different sample sites and ellipses indicate 95% confidence interval.

**Figure 3:**
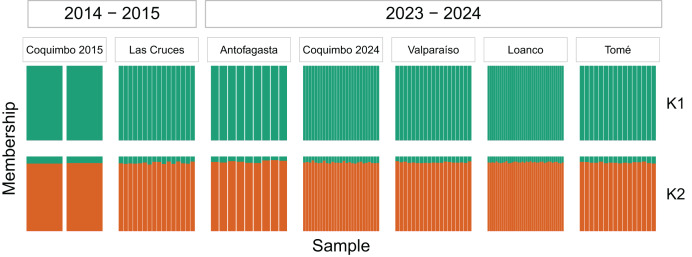
Genetic structure of *Cancer porteri* using STRUCTURE software. Structure inferred from 3,532 neutral SNPs on samples from 2014–2015 and 2023–2024. Results showed K = 1 and K = 2. Vertical lines represent individuals and colors represent the genetic cluster. The length of colored segments represents the probability of belonging to each genetic cluster.

Both principal coordinates analysis (PCoA) and STRUCTURE analysis shows a lack of genetic structure across the sample locations and periods. No clustering was obtained in the PCoA, grouping all samples independent of location and sampling year (Fig. 2). Results of the STRUCTURE analysis show that the posterior probability of K = 2 is higher than K = 1 (mean LnP(K) = −157,150.68 for K = 2 and −157,396.88 for K = 1), but the individual assignment to each K group in the bar plots indicates that the most biologically relevant case is K =1 (Fig. 3). Contemporary *N*_*e*_ estimates indicate large effective sizes for the entire genetic population in both periods. In 2014-2015 the estimate of *N*_*e*_ was 9,881.7 (parametric 95% CI [1,361.7 – ∞]; JackKnife 95% CI [384.1 – ∞]), while in 2023-2024 was 2,811.9 (parametric 95% CI [2,105.7–4,217.0]; JackKnife 95% CI [1,520.4–17,110.7]).

**Figure 4:**
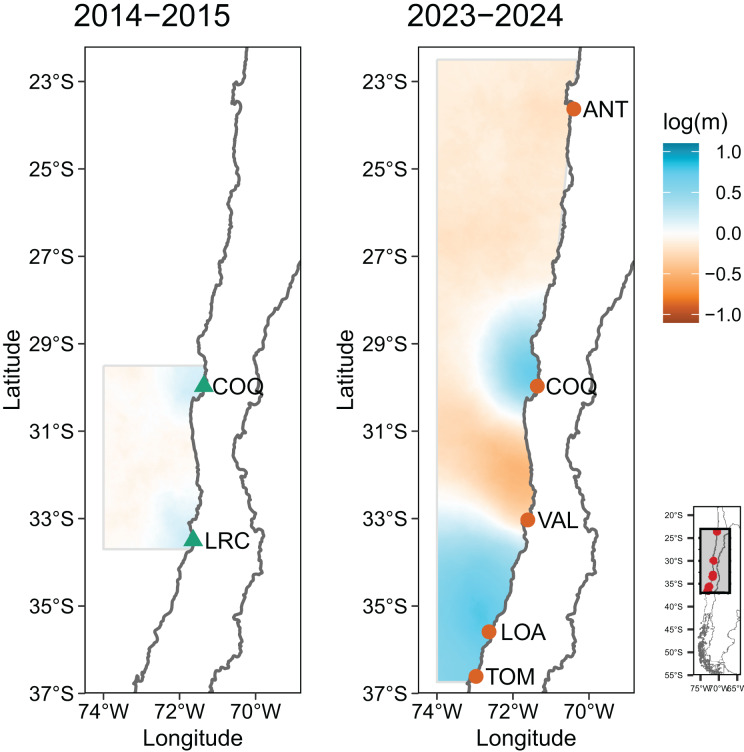
Estimated Effective Migration Surfaces (EEMS) of *C. porteri*. Analysis performed considering 2,196 (2014–2015) and 3,513 (2023–2024) neutral SNPs. Colors represent relative rates of migration, ranging from lower than average (orange) to higher than average (blue). Log(m) denotes the effective migration rate on a log_10_ scale, relative to the overall migration rate across the sample area, thus log(m) = 1 is an effective migration ten times greater than the average, and a log(m) = −1 ten times less than the average. ANT, Antofagasta; COQ, Coquimbo; LRC, Las Cruces; VAL, Valparaíso; LOA, Loanco; TOM, Tomé.

Gene flow analysis reveals that migration occurs between all sample sites. Historical migration analysis with MIGRATE under different models of migration (full, panmictic and northward) shows that the full model is most appropriate for 2014–2015 and 2023–2024 in both datasets of 1,000 SNPs tested (Table 3), meaning that migrations occur between all sites studied, in both years. Mean migration rates values were similar in all directions and in both data sets, although estimations for 2014–2015 were higher than for 2023–2024. In 2014–2015, the values ranged between 449.0 to 459.1, and in 2023–2024 the values ranged between 196.8 to 290.1 (Table 4). The analysis of the effective migration surfaces (EEMS) supports the results obtained by the other methods pointing out the presence of migrants among all sites within both sampling periods, with values close to the mean migration, but with higher deviations in the period 2023–2024, indicating stronger patterns of migration in this period (Fig. 4). Interestingly, the area between 30 to 33°S of latitude, between Coquimbo and Valparaíso—Las Cruces sampling sites, is an area with lower-than-average migration.

**Table 3:** Bezier approximated score for each model tested with the MIGRATE software. Analysis was performed on two datasets (A and B) of 1,000 random SNPs for the 2014–2015 and 2023–2024 samples. Full, full independent migration between sample sites; Panmixia, panmictic model; Northward, migration only occurs towards the north. The Full model is the most appropriate in all data sets, with the highest Bezier score.

Model	Log(mL)			
	2023–2024		2014–2015	
	Dataset A	Dataset B	Dataset A	Dataset B
Full	2,624.05	2,663.93	−1,372.51	−1,411.16
Panmixia	−6,321.28	−6,363.38	−1,730.12	−1,769.08
Northward	−3,828.16	−3,936.49	−1,676.42	−1,705.82

**Table 4:** MIGRATE results considering the full independent migration between sample sites. Mean mutation-scaled effective population size (*θ*) for each sample site and mean mutation-scaled effective migration rate (*M*) between all pairs of sites determined with a full migration model in MIGRATE software for two datasets (A and B) of 1,000 random SNPs for the 2014–2015 and 2023–2024 samples.

Year	Parameter	Site/Direction	Dataset A	Dataset B
2014–2015	Mean *θ*	Coquimbo	0.04306	0.04174
		Las Cruces	0.0492	0.04778
	Mean *M*	Las Cruces to Coquimbo	459.0815	450.1357
		Coquimbo to Las Cruces	456.28	449.0055
2023–2024	Mean *θ*	Antofagasta	0.07289	0.07115
		Coquimbo	0.07947	0.0805
		Valparaíso	0.0757	0.07581
		Loanco	0.08551	0.08959
		Tomé	0.0737	0.07578
	Mean *M*	Coquimbo to Antofagasta	256.3224	241.0506
		Valparaíso to Antofagasta	214.1501	284.1324
		Loanco to Antofagasta	228.5703	249.087
		Tomé to Antofagasta	263.5533	246.1382
		Antofagasta to Coquimbo	264.1587	290.711
		Valparaíso to Coquimbo	232.2781	218.6719
		Loanco to Coquimbo	226.4491	231.3014
		Tomé to Coquimbo	234.7453	211.6124
		Antofagasta to Valparaíso	238.3435	272.6791
		Coquimbo to Valparaíso	246.7014	226.691
		Loanco to Valparaíso	267.589	241.2887
		Tomé to Valparaíso	277.4567	196.8232
		Antofagasta to Loanco	226.5032	210.8334
		Coquimbo to Loanco	214.7834	227.0839
		Valparaíso to Loanco	224.7039	211.8586
		Tomé to Loanco	254.0484	202.2428
		Antofagasta to Tomé	232.6666	221.6178
		Coquimbo to Tomé	239.0149	218.9749
		Valparaíso to Tomé	270.3821	238.8804
		Loanco to Tomé	273.2603	248.3736

## Discussion

The analysis of the genetic structure of *Cancer porteri* revealed a lack of population genetic structure across a geographic distance of 1,500 km and across a temporal scale of 10 years, showing evidence of a single population between Antofagasta and Tomé in both sampling periods. This indicates that there is spatial stability and absence of detectable temporal differentiation in the population of this species in the area and period studied. This lack of genetic structure appears to be related to the high gene flow estimated between all locations studied. Our estimates showed gene flow in all sampling sites, as was revealed by the different models in the MIGRATE software. This high gene flow is maintained even in the presence of an area between 30 to 33°S of latitude with lower-than-average effective migration, which coincides with the presence of a biogeographic break at the 30°S.

### Null spatial genetic structure

In the South Pacific coast, there is a relationship between PLD and genetic structure in marine invertebrates, with species with PLD less than a month presenting genetic discontinuities (Haye et al., 2014). Consequently, previous studies have found no genetic structure in other crustacea with a PLD of over 1 month. Specifically, two other cancrids in the same area have been described with the same pattern. In *M. edwardsii*, there is no genetic structure between the 33 and 45°S, described using both SNPs and microsatellites as molecular markers (Rojas-Hernandez et al., 2016; Veliz et al., 2022). Similar is the case for *R. setosum* using AFLP (Gomez-Uchida et al., 2003) and 2,383 SNPs (Tubin-Arenas et al., 2025). Globally, most cancrids populations encompass large oceanic areas without an apparent genetic structure (Tubin-Arenas et al., 2025). Therefore, *C. porteri* is expected to constitute a single large population throughout its entire range, although this should be confirmed with samples from Perú.

The lack of genetic structure in the studied range indicates that the biogeographic break described at 30°S (Camus, 2001) doesn’t affect gene flow of *C. porteri*, even if effective migration is lower-than-average south of this latitude in both sets of years. Other invertebrates with long PLD (>30 days) along the Humbolt Current System are also unaffected by this break, including the echinoderms *Heliaster helianthus* and *Arbacia nigra* (Haye et al., 2014), the gastropod *Concholepas concholepas* (Cárdenas, Castilla & Viard, 2009), and the crustaceans *Emerita analoga*, *Grimothea monodon* and *Jehlius cirratus* (Zakas et al., 2009; Haye et al., 2010, 2014), although all of these studies used cytochrome c oxidase subunit I (COI) as genetic marker, which has shown less capacity to detect finer genetic structure patterns compared to SNPs (Segovia et al., 2017).

### Absence of detectable temporal differentiation in genetic variability

The species showed an absence of detectable temporal differentiation in its genetic structure. Although the sampling includes only two locations from 2014–2015 and a 10-year interval that spans multiple generations based on the generation time of 2.3 years (IFOP, 2017), the results suggest that the genetic structure remained unchanged during the period examined. In the case of the crab *M. edwardsii* in Chile, genetic variability described with SNPs showed no changes between samples obtained in 2013–2014 and 2020–2021 (Veliz et al., 2022), and in settling individuals in the locality of Los Molinos (Valdivia) from 2009 to 2012 using microsatellites (Rojas-Hernandez et al., 2016). Although some differences in genetic diversity parameters were found between 2014–2015 and 2023–2024 in this study, these small differences are probably due to the smaller sample sizes of 2014–2015 samples. Crabs in other regions also have shown a lack of temporal genetic differentiation, for example, *Carcinus maenas* in the Iberian Peninsula (Domingues et al., 2010, 2011), the crab *Cancer pagurus* in Norway (Ungfors et al., 2009), the Pacific Geoduck Clam *Panopea generosa* (Vadopalas, Pietsch & Friedman, 2010) and the Arctic surf clam *Mactromeris polynyma* (Cassista & Hart, 2007).

Determining whether genetic temporal differentiation exists in fishery resources is an important precedent for their management. For example, in the Chilean mussel *Mytilus chilensis*, small but significant temporal genetic structure may be associated with temporal variations in the availability of spat in natural beds (Haye & Segovia, 2023). Although there have been no studies on stock size variation across time of *C. porteri*, its landings have presented high variation over the last 25 years. These variations probably correspond to suboptimal landing reports for brachyurans (Muñoz et al., 2006), so these reports are inadequate to make inferences about stock size changes across time. Future studies should consider multiple years of sampling and a broader time range to confirm that the genetic structure of *C. porteri* is stable across generations.

The large *N*_*e*_ estimates for both years are consistent with the genetic evidence that cancrid crabs typically form large, well connected metapopulations across their geographical ranges (Tubin-Arenas et al., 2025). Although point estimates decreased from 9,881.7 in 2014–2015 to 2,811.9 in 2023–2024, the broad confidence intervals, particularly the jackknife estimates extending to infinity, indicate no significant change in *N*_*e*_ between periods. Similar *N*_*e*_ estimates have been reported for other brachyurans. In the blue crab *Callinectes sapidus*, populations from various regions along the western Atlantic coast, from the United States to Brazil, all exhibit *N*_*e*_ estimates of infinite size (Macedo et al., 2019). In Australia, populations of *Portunus armatus* have large *N*_*e*_ on the east coast and infinite estimates for western and northern populations (Premachandra et al., 2023). The cancrid *M. edwardsii* showed infinite *N*_*e*_ when megalopa samples from four consecutive years were analyzed in southern Chile (Rojas-Hernandez et al., 2016). Effective population size estimates extending to infinity are a known limitation of methods for estimating *N*_*e*_ in large populations, as small sample sizes relative to large populations do not provide sufficient information to determine the upper bounds of *N*_*e*_, although they still provide useful estimates for lower bounds (Waples & Do, 2010).

### Gene flow

Migration analyses detected high gene flow across all sites, which explains the lack of genetic structure, which is consistent with previous works done on other species of Cancridae. Both *M. edwardsii* and *R. setosum* showed high gene flow across 1,700 and 2,700 km of coast, respectively (Veliz et al., 2022; Tubin-Arenas et al., 2025). The long PLD of Cancridae probably explains the high gene flow of this species, and although PLD is correlated with dispersal distance, it alone may overestimate dispersal potential and only explain about 50% of the variance in dispersal ability, with other factors such as larval behavior largely influencing larval dispersal (Shanks, 2009). There have been no studies specifically on the behavior of *C. porteri* larvae to better comprehend the factors that contribute to their high dispersal. However, several studies have examined larval dispersal patterns in other decapods and members of the Cancridae family. The larvae of the Dungeness crab *Cancer magister* in the California current migrate offshore beyond the continental shelf during the early developmental stages, later returning to shore for settlement after reaching the megalopa stage. This process is mediated by oceanographic events such as tidal currents (Miller & Shanks, 2004), onshore movement of deep water driven by upwelling (Shanks & Roegner, 2007), and vertical migration patterns (Park & Shirley, 2005). These mechanisms together promote high connectivity among local populations, except for those inhabiting fjord systems (O’Malley et al., 2017). A similar pattern has been described in the gulf of Arauco, Chile, for larvae of subtidal decapods including *Neotrypaea uncinata* (Yannicelli et al., 2006a). Other study in the same gulf indicated that larvae of *Cancer* spp. interact with oceanographic flows, showing no vertical migration pattern during upwelling periods, remaining in surface waters above the pycnocline in high oxygenated waters to facilitate transport offshore from the gulf (Yannicelli et al., 2006b).

In northern Chile, similar patterns have been described that favor transport offshore. Between 20°40′S and 21°20′S early larvae of *Cancer* spp. are distributed more widely in the horizontal plane than in the vertical, being active migrants in surface waters above the pycnocline and highly exposed to advection caused by upwelling. Later stages (after zoea IV) return to waters closer to the coast (Rivera, Guzmán & Palma, 2019). Other studies also report that larvae can reach several km offshore. Between Arica and Huasco (18–28°S) and under the influence of the 1985 El Niño Southern Oscillation, Cancridae larvae were more abundant close to the coast (5 km offshore), but in one location (19.5°S) were found 140 km offshore (Baez & Martin, 1992). In a later study describing decapod larvae distribution between Caldera and Easter Island Caldera (27°S), Cancridae larvae were more abundant between 56 to 520 km from the coast, associated to cold water and low salinities from the Humbolt current that is present in the 1,000 km from the coast and between the 0 and 100 m of depth (Rivera & Mujica, 2004). All this evidence on the larval distribution of Cancridae species, both in the northern and in the south-central part of Chile, support the conclusion of a high larval dispersal that would allow for substantial gene flow along the coast, although further studies are needed on the larval behavior of *C. porteri* throughout its larval development in order to better understand its migration patterns and ability to disperse in the water column.

### Oceanographic considerations

The physical oceanography of the Humboldt Current System likely could play a critical role in the dispersal and genetic mixing of benthic species, such as crabs. The Humboldt Current System (HCS) along the Chilean coast is characterized by a persistent bidirectional flow, composed of the northward-flowing Humboldt Current (HC) at the surface, and two southward-flowing currents: the Peru-Chile Countercurrent (PCC), which is superficial, and the Peru-Chile Undercurrent (PCUC), which is subsurface flows from the eastern tropical Pacific to at least as far south as 48°S (Nelson Silva & Neshyba, 1979; Strub et al., 1998; Pizarro et al., 2002; Chevallier et al., 2021). The HC flows near the surface year-round, while the PCC flows southward above approximately 50–100 m depth, and the PCUC flows southward at subsurface depths around 50 and 300 m, intensifying and shoaling during the austral summer (Pizarro et al., 2002; Chevallier et al., 2021). This circulation system creates conditions for bi-directional alongshore transport, which can significantly affect the dispersal potential of marine species with planktonic larval stages occurring between 20 and 45°S. Early life stages may be transported northward by the surface HC, while southward dispersal may occur *via* the PCC or deeper PCUC, depending on larval depth or ontogenetic vertical migration. These dynamics likely could enhance gene flow and promote genetic mixing.

In addition, based on observational data, the mean flow of the current velocity of the Humboldt current can be estimated at approximately 0.1 m/s (Huyer et al., 1991; Shaffer et al., 1999; Fuenzalida et al., 2008). At this speed, a crab passively transported by the current could travel nearly 777 km over a 90-day pelagic duration, which falls within the larval period reported for several decapod species. This distance corresponds to roughly seven degrees of latitude, depending on the location along the Chilean coast. Such a potential for long-distance dispersal *via* surface or subsurface currents suggests that larvae could connect populations across broad spatial scales. These combined oceanographic and biological processes may help explain the lack of significant genetic differentiation observed in *C. porteri* and other crab species distributed along the southeastern Pacific coast.

### Future considerations for fishery management

A group of commercially important invertebrates in the Southern Pacific Ocean exhibit no population genetic structure, including the cephalopods *Octopus mimus* (Palacios-Farías et al., 2025) and *Dosidicus gigas* (Sandoval-Castellanos, Uribe-Alcocer & Díaz-Jaimes, 2010), the gastropod *Concholepas concholepas* (Cárdenas, Castilla & Viard, 2016), and the crabs *M. edwardsii* (Rojas-Hernandez et al., 2016; Veliz et al., 2022) and *R. setosum* (Tubin-Arenas et al., 2025), all of which are exploited at the artisanal level. In Chile, crab fisheries are regulated by general measures such as minimum landing size and a ban on harvesting ovigerous females. However, they remain less prominent than other fisheries despite their local economic relevance and potential for growth (IFOP, 2017). In the case of *C. porteri*, consumption is limited to its chelae due to the thinness of its pereiopods, leading to the common practice of removing chelae and returning the individuals to the sea (IFOP, 2017). This practice may affect reproduction, as males typically form harems of 3–5 females and provide protection to them (Jesse & Stotz, 2002), so males lacking or with reduced chelae may compromise reproductive success. Although our results suggests that such practices have not yet altered the population, likely due to the species’ large effective population size, high dispersal capacity, and reproductive potential, future research should aim to integrate genetic, demographic, and biological approaches to detect biological patterns that are informative for biological resources management (Mariani & Bekkevold, 2014), particularly in the southern part of *C. porteri* distribution where extraction is concentrated.

## Conclusion

Our results indicate that *C. porteri* constitutes a single genetic population in the area between Antofagasta and Tomé (1,500 km), with no genetic structure, uniform values of genetic diversity, a large effective population size and high gene flow across all sample sites, that is also stable in a 10-year period. The species long PLD, high fecundity and high advection capacity offshore likely contribute to a high dispersal potential and connectivity. Future research should incorporate samples from the northernmost portion of the species’ range to confirm whether *C. porteri* forms a single panmictic population throughout its entire distribution. Additionally, it is necessary to incorporate demographic studies to define fishery stock to better inform management strategies. Without this information, it remains difficult to assess whether *C. porteri* is vulnerable to local demographic fluctuations and fishing pressure, emphasizing the need to integrate genetic and demographic information for the sustainable management of this species.

## Supplemental Information

10.7717/peerj.20727/supp-1 Supplemental Information 1 Information for samples obtained from the SCBUCN collection.

## Additional Information and Declarations

### DNA Deposition

The following information was supplied regarding the deposition of DNA sequences:

The data is available at the Sequence Read Archive (SRA): PRJNA1303652.

### Data Availability

The following information was supplied regarding data availability:

The code and raw data are available at Figshare: Soto, Juan (2025). High gene flow and lack of genetic structure in the commercially important crab *Cancer porteri* (Brachyura: Cancridae) along 1,500 km of the Chilean coast revealed by SNP markers. figshare. Dataset. https://doi.org/10.6084/m9.figshare.29591624.v2.
