# Supplementary material for: High gene flow and lack of genetic structure in the commercially important crab Cancer porteri (Brachyura: Cancridae) along 1,500 km of the Chilean coast revealed by SNP markers
Source: PeerJ. 2026 Jan 22;14:e20727. doi: 10.7717/peerj.20727 (PMC12832060; doi:10.7717/peerj.20727)
Supplement: Supplemental Information 1 [file peerj-14-20727-s001.docx]

**Supplemental Table S1. Information for samples obtained from the SCBUCN collection.**

| trayCode | catalogNumber | Date | Site | Latitude | Longitude |
| --- | --- | --- | --- | --- | --- |
| BBF1C2N4 | SCBUCN-3197 | 2009-11-23 | Coquimbo | -30.3816 | -71.9627 |
| BBF1C4N3 | SCBUCN-5649 | 2015-10-21 | Coquimbo | -29.8252 | -71.3196 |
| BAF1C8N2 | SCBUCN-9282 | 2023-11-27 | Antofagasta | -23.2933 | -70.6166 |
| BCF1C5N5 | SCBUCN-9292 | 2023-11-26 | Antofagasta | -24.2211 | -70.5376 |
| BDF1C5N5 | SCBUCN-9323 | 2023-11-25 | Antofagasta | -24.8865 | -70.5430 |
| BBF1C8N2 | SCBUCN-9340 | 2023-11-27 | Antofagasta | -23.7573 | -70.4933 |
| BDF1C5N5 | SCBUCN-9385 | 2023-11-24 | Antofagasta | -25.6883 | -70.9981 |
| BDF1C5N5 | SCBUCN-9393 | 2023-11-25 | Antofagasta | -25.0888 | -70.5278 |
| BDF1C5N5 | SCBUCN-9395 | 2023-11-25 | Antofagasta | -25.0888 | -70.5278 |
| BCF1C8N2 | SCBUCN-9438 | 2023-11-28 | Antofagasta | -22.7410 | -70.3148 |
| BCF1C8N2 | SCBUCN-9439 | 2023-11-28 | Antofagasta | -22.7410 | -70.3148 |
| BCF1C8N2 | SCBUCN-9440 | 2023-11-28 | Antofagasta | -22.7410 | -70.3148 |
| BBF1C8N2 | SCBUCN-9465 | 2023-11-26 | Antofagasta | -24.6041 | -70.5818 |
